# PhoU interaction with the PhoR PAS domain is required for repression of the *pho* regulon and *Salmonella* virulence, but not for polyphosphate accumulation

**DOI:** 10.71150/jm.2505013

**Published:** 2025-09-30

**Authors:** Seungwoo Baek, Soomin Choi, Yoontak Han, Eunna Choi, Shinae Park, Jung-Shin Lee, Eun-Jin Lee

**Affiliations:** 1 Department of Life Sciences, School of Life Sciences and Biotechnology, Korea University, Seoul 02841, Republic of Korea; 2 Department of Molecular Bioscience, College of Biomedical Science, Kangwon National University, Chuncheon 24341, Republic of Korea

**Keywords:** phosphate transport, mouse virulence, polyphosphate, histidine kinase

## Abstract

The *pho* regulon plays a critical role in maintaining phosphate homeostasis in bacteria, with the PhoU protein functioning as a regulator that bridges the PhoB/PhoR two-component system and the PstSCAB_2_ phosphate transporter. While PhoU is known to suppress PhoR autophosphorylation under high phosphate conditions via interaction with its PAS domain, its broader regulatory functions remain elusive. Here, we investigated the role of the PhoU Ala147 residue in *Salmonella enterica* serovar Typhimurium using a *phoU*^A147E^ substitution mutant. Bacterial two-hybrid and immunoprecipitation assays confirmed that Ala147 is essential for PhoU-PhoR PAS domain interaction, and its substitution leads to derepression of *pho* regulon genes, even in high phosphate conditions. This disruption impaired *Salmonella* survival inside macrophages and mouse virulence, demonstrating the importance of PhoU-PhoR interaction in *Salmonella* pathogenesis. However, unlike the *phoU* deletion mutant, the *phoU*^A147E^ mutant does not exhibit growth defects or polyphosphate accumulation, indicating that the PhoU-PhoR interaction is not involved in these phenotypes. Our findings reveal PhoU as a multifaceted regulator, coordinating phosphate uptake and *pho* regulon expression through distinct molecular interactions, and provide new insights into its role in bacterial physiology and virulence.

## Introduction

**Fig. 1.**
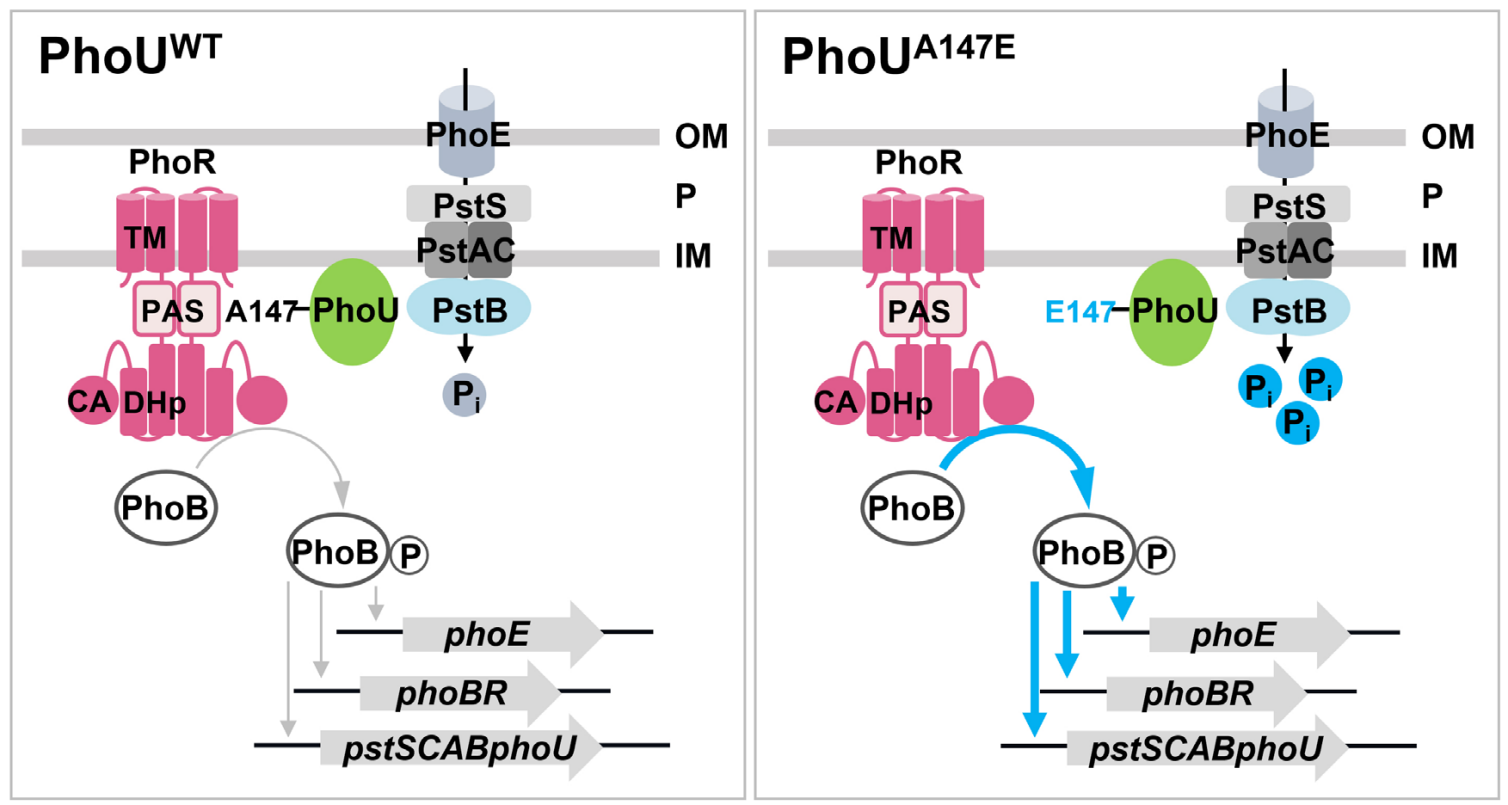
Regulatory role of PhoU in *Salmonella*. Under low-phosphate conditions, the histidine kinase PhoR undergoes autophosphorylation at a conserved histidine residue in its DHp domain and subsequently transfers the phosphate to the response regulator PhoB. Phosphorylated PhoB binds to promoter regions and activates the expression of the *pho* regulon, which includes the PhoB/PhoR two-component system itself, the high-affinity phosphate transporter PstSCAB_2_, and the negative regulator PhoU. PhoU interacts with both PhoR and the ATPase subunit PstB of the PstSCAB_2_ transporter to inhibit PhoR kinase activity under high-phosphate conditions. The Ala147 residue of PhoU mediates its interaction with the PAS domain of PhoR. Substitution of Ala147 with glutamate (PhoU A147E) disrupts this interaction and constitutively activates *pho* regulon expression, independent of phosphate availability.

The *pho* regulon plays an important role in regulating homeostasis of inorganic phosphate (Pi), one of the essential molecules in biology (Hsieh and Wanner, 2010). The core components of this regulon include *phoB/phoR*, *pstSCAB*, *phoE*, and *phoU*, which encode the PhoB/PhoR two-component system, the PstSCAB_2_ high-affinity phosphate transporter, the PhoE outermembrane porin, and the PhoU regulatory protein, respectively (Fig. 1). The PhoB/PhoR two-component system controls the signalling pathway of the *pho* regulon and is highly conserved in both Gram-negative and Gram-positive bacteria (Glover et al., 2007; Hsieh and Wanner, 2010; Lamarche et al., 2008). In this system, PhoR is a membrane-bound histidine kinase that senses phosphate availability and activates its response regulator, PhoB. PhoR contains three functional domains: a Per-Arnt-Sim (PAS) domain, a dimerization and histidine phosphotransfer (DHp) domain, and a catalytic active/ATP-binding (CA) domain (Lamarche et al., 2008). In a phosphate-limiting condition, PhoR auto-phosphorylates a histidine residue within its DHp domain and activates PhoB by transferring a phosphoryl group (Ellison and McCleary, 2000; Hsieh and Wanner, 2010; Makino et al., 1996). When the PhoB response regulator is phosphorylated, it binds to the promoter region known as the Pho box and activates PhoB-dependent gene expression (Bachhawat et al., 2005; Gao and Stock, 2015; Makino et al., 1988). By contrast, in a high phosphate condition, the expression of the *pho* regulon is repressed to maintain Pi homeostasis.

In this phosphate signalling, PhoU primarily functions as a negative regulator, as indicated by the observation that *phoU* deletion increases PhoR autophosphorylation and the expression of *pho* regulon genes regardless of phosphate levels (de Almeida et al., 2015; Lubin et al., 2016; Steed and Wanner, 1993; Wang et al., 2013). In addition, *phoU* seems to function as a molecular bridge in phosphate signalling pathway. Unlike other sensor kinases that directly recognize and respond to the environmental changes through periplasmic domains, PhoR does not possess a periplasmic domain possibly sensing extracellular phosphate concentrations. Instead, it is proposed that PhoR kinase activity is tightly linked to the activity of the PstSCAB_2_ transporter (Piepenbreier et al., 2017). PhoU seems to be central in this regulation, because PhoU physically binds both the PAS domain of PhoR and PstB of PstSCAB_2_ transporter (Gardner et al., 2014), suggesting that PhoU functions as a molecular bridge connecting the PhoB/PhoR two-component system and the PstSCAB_2_ phosphate transporter (Gardner et al., 2014, 2015) (Figs. 1 and S1E). PhoU binding to the PhoR PAS domain is believed to be essential for suppressing PhoR kinase activity in high phosphate conditions, based on the observation that *phoU* deletion derepresses *phoB*-dependent gene expression in both high and low phosphate conditions (de Almeida et al., 2015; Lubin et al., 2016; Steed and Wanner, 1993; Wang et al., 2013). Similarly, it is assumed that activation of PhoR kinase in low phosphate is mediated by PhoU’s detachment from the PAS domain of PhoR; however, this mechanism remains unclear.

Recently, PhoU was proposed to function as a dual regulator in phosphate signalling. In *Salmonella enterica* serovar Typhimurium, PhoU interacts with PhoR via not only the PAS domain but also the CA domain (Choi et al., 2022). Amino acid substitution studies have shown that PhoU interaction through the CA domain is required for activating the expression of phosphate transport genes in low Mg^2+^ medium, an infection-relevant condition. Given that the PhoU-PhoR PAS domain interaction is involved in repression of phosphate signalling in a high phosphate condition, the PhoU protein appears to modulate phosphate signalling differently in high phosphate and low Mg^2+^ conditions via distinct interactions. Therefore, PhoU’s role in controlling PhoR histidine kinase activity needs to be dissected more carefully. Additionally, PhoU likely serves as a link between the phosphate-sensing PhoB/PhoR two-component system and the potassium-sensing KdpE/KdpD two-component system (Schramke et al., 2017), as PhoU enables the KdpE-dependent expression of potassium transporter genes in the absence of the KdpD histidine kinase, in response to phosphorylation initiated by the PhoR histidine kinase (Schramke et al., 2017).

Recent studies have further established that PhoU is a multifaceted regulator involved in bacterial signalling and physiology (Baek and Lee, 2024). Interestingly, *phoU* mutations lead to the accumulation of polyphosphate (polyP) granules in several bacterial species (de Almeida et al., 2015; Li and Zhang, 2007; Lubin et al., 2016; Morohoshi et al., 2002). Although both polyphosphate accumulation and derepression of the *pho* regulon are linked to *phoU* deletion, these phenotypes do not always co-occur. In *Caulobacter crescentus*, for example, PhoU has been reported to promote polyphosphate accumulation without repressing *pho* regulon signalling in high-phosphate conditions (Lubin et al., 2016). Polyphosphate accumulation is also associated with various phenotypes in other bacteria. In *Staphylococcus epidermidis* and *Mycobacterium marinum*, *phoU* deletion leads to polyphosphate accumulation and exhibits impaired stress resistance and persister formation (Wang et al., 2013, 2017). Moreover, in *Streptomyces*, *phoU* deletion accumulates polyphosphate and decreases secondary metabolite biosynthesis.

Although PhoU has been implicated in regulating diverse cellular functions as described above, the mechanism by which a single protein governs such a wide range of phenotypes remains unclear. In particular, how PhoU interacts with and influences other proteins is not well understood. To address this, we investigated how a specific amino acid substitution in PhoU disrupting the PhoR PAS domain interaction contributes to its regulatory functions. By comparing the phenotypes of a *phoU* deletion mutant and a PhoU A147E-substituted mutant, we found that not all Δ*phoU*-associated phenotypes result from disruption of the PhoU-PhoR PAS domain interaction. This genetic dissection revealed that PhoU-PhoR PAS domain interaction is involved in repressing the *pho* regulon in high phosphate conditions and contributes to *Salmonella* pathogenesis. However, the same interaction is not involved in maintaining normal growth rate or preventing polyphosphate accumulation, suggesting that these phenotypes result from different molecular mechanisms.

## Materials and Methods

### Bacterial strains, plasmids, primers, and culture conditions

The bacterial strains and plasmids used in this study are listed in Table S1. All *Salmonella enterica* serovar Typhimurium strains are derived from the wild-type strain 14028s and were constructed by phage P22-mediated transductions. DNA oligonucleotides are also listed in Table S1. Bacteria were grown at 37°C in Luria-Bertani broth (LB), and N-minimal media (pH 7.7) supplemented with 0.1% casamino acids, 38 mM glycerol, and the indicated concentrations of MgCl_2_. For low-phosphate N-minimal media, 10 mM KH_2_PO_4_ in the N-minimal media was replaced by 0.01 mM KH_2_PO_4_. *Escherichia coli* DH5α was used as the host for the preparation of plasmid DNA, and BTH101 lacking the *cya* gene was used as the host for the bacterial two-hybrid system. Ampicillin was used at 50 μg/ml, chloramphenicol at 25 μg/ml, kanamycin at 50 μg/ml, or tetracycline at 10 μg/ml. IPTG (isopropyl β-D-1-thiogalactopyranoside) was used at 0.25 mM, L-arabinose at 1 mM, and X-Gal (5-bromo-4-chloro-3-indolyl β-D-galactopyranoside) at 80 μM.

### Plasmid construction

For the plasmid pBAD33-*phoU*^A147E^-His was constructed as follows: a PCR fragment corresponding to the *phoU*^A147E^ gene was generated by PCR with the primer pairs KHU767/KHU768 using SM323 genomic DNA as a template. The amplified DNA fragment was digested with XbaI and HindIII and cloned into pBAD33 digested with the same enzymes.

### Construction of *phoU*^A147E^ strain with a C-terminal Myc-tagged version of the *phoR* gene at its native chromosomal locus

*Salmonella* *phoU*^A147E^ strain with a C-terminal myc-tag fused to the *phoR* gene was generated by the PCR-based tandem epitope tagging system (Cho et al., 2006). A Km^R^ cassette for the *phoR*-8×myc gene was PCR amplified from plasmid pBOP508 using KU452 and KU453 primers. The resulting PCR product was integrated into the SM323 chromosome to generate SM443 (*phoU*^A147E^, *phoR*-8×myc::Km^R^). The SM457 (*phoU*^A147E^, *phoR*-8×myc) strain was generated by removing the Km^R^ cassette from SM443 using plasmid pCP20 as described (Datsenko and Wanner, 2000).

### Bacterial two hybrid (BACTH) assay

To assess protein (or peptide)-protein interactions in vivo, a bacterial two-hybrid (BACTH) assay was conducted as described (Karimova et al., 1998). The *Escherichia coli* BTH101 strain was co-transformed with derivatives of the pUT18, pUT18C, and pKT25 plasmids. The strains were grown overnight at 37°C in LB supplemented with ampicillin (50 μg/ml) and kanamycin (50 μg/ml). Then, 4 μl of cells was spotted on LB agar plate with 500 μM IPTG, 100 μM ampicillin, 100 μM kanamycin, and 80 μM X-Gal for 48 h at 30°C.

### Immunoprecipitation assay

The interaction between the PhoU and PhoR proteins was investigated in wild-type *Salmonella* expressing the *phoU* gene from an arabinose-inducible plasmid (pBAD33-*phoU*-His and pBAD33-*phoU*^A147E^-His) and a C-terminally gfp-tagged *phoR* gene or its derivatives (pTGFP-*phoR*), or the empty vector (pTGFP) from the constitutive P_lac_ promoter.

Cells were grown overnight in N-minimal medium containing 10 mM Mg^2+^, then diluted 1:100 into 15 ml of fresh N-minimal medium containing 10 mM Mg^2+^ and incubated for 3 h. Cells were washed and resuspended in 15 ml of N-minimal medium containing 0.5 mM Mg^2+^, 1 mM L-arabinose and 0.25 mM IPTG, then incubated for an additional 1 h. After normalizing cell density by measuring OD_600_, crude extracts were prepared by sonication in Tris-buffered saline (TBS). For the pull-down assay using anti-GFP antibodies, 50 μl of crude extract was reserved as input. The remaining 400 μl was incubated with 25 μl of GFP-Trap^®^_A beads (Chromotek) for 1 h at 4°C on a nutator. The beads were washed twice with TBS and collected by centrifugation. Bound proteins were eluted with SDS sample buffer. The protein extracts were separated on 12% SDS-polyacrylamide gels, transferred onto nitrocellulose membranes, and analyzed by Western blot. Membranes were incubated overnight at 4°C with primary antibodies against His-tag (rabbit, 1:5,000; Rockland^TM^, 600-401-382S) and GFP (rabbit, 1:2,500; Rockland^TM^, 600-401-215). After washing, blots were incubated for 1 h with HRP-conjugated anti-rabbit IgG secondary antibody (1:10,000; ThermoFisher, 31460). Detection was performed using the SuperSignal^®^ West Femto Maximum Sensitivity Substrate (ThermoFisher).

### Western blot analysis

Cells were cultured for 5 h in 15 ml of N-minimal medium supplemented with either 10 mM or 0.01 mM inorganic phosphate (Pi). After incubation, cell density was normalized by measuring OD_600_. Crude extracts were prepared by sonication in Tris-buffered saline (TBS). Proteins were separated on a 12% SDS–polyacrylamide gel and transferred to nitrocellulose membranes. Fur and Myc-tagged proteins were detected using anti-Fur (rabbit, 1:5,000) and anti-Myc (mouse, 1:5,000; MBL, M192-3) primary antibodies. Membranes were incubated overnight at 4°C with these primary antibodies. Subsequently, the blots were incubated for 1 h at room temperature with horseradish peroxidase (HRP)-conjugated secondary antibodies: anti-rabbit IgG (ThermoFisher, 31460) or anti-mouse IgG (Sigma-Aldrich, NA931V), each at a 1:10,000 dilution. Signal was detected using the SuperSignal^®^ West Femto Maximum Sensitivity Substrate (ThermoFisher, 34095).

### Real-time quantitative reverse transcription PCR (qRT-PCR)

Bacteria were cultured for 5 h in 15 ml of N-minimal medium containing either 10 mM or 0.01 mM inorganic phosphate (Pi). Total RNA was extracted using the RNeasy Kit (QIAGEN) following the manufacturer’s instructions. The purified RNA was quantified using a NanoDrop spectrophotometer (NanoDrop Technologies). Complementary DNA (cDNA) was synthesized using the PrimeScript^TM^ RT reagent Kit (TaKaRa). The mRNA levels of *phoU* and *phoE* were quantified by real-time PCR using SYBR Green PCR Master Mix (TOYOBO) and specific primers (*phoU*: KHQ097/KHQ098, *phoE*: KHQ015/KHQ016). Reactions were performed on a StepOnePlus^TM^ Real-Time PCR system (Applied Biosystems, USA). Transcript levels were calculated based on a standard curve generated from *Salmonella enterica* serovar Typhimurium 14028s genomic DNA of known concentration (Bong et al., 2024). Expression levels were normalized to 16S ribosomal RNA using primers 6970 and 6971.

### Structural modeling of the PhoR–PhoU–PstSCAB_2_ complex

Structural predictions of the PhoR-PhoU-PstB within the PhoR–PhoU–PstSCAB_2_ complex were generated using AlphaFold3. Amino acid sequences of *Salmonella enterica* serovar Typhimurium 14028s PhoU (UniProt: A0A0F6B922), PhoR (A0A0F6AXL9), and PstB (A0A0F6B923) were retrieved from the UniProt database. Protein complex structures were predicted using the AlphaFold-Multimer implementation, which accounts for protein-protein interaction interfaces (Abramson et al., 2024). Modeling was performed using default parameters, and the highest-ranked prediction was selected based on the predicted TM-score (pTM = 0.71) and the inter-chain predicted TM-score (ipTM = 0.69). This model was then used for interface analysis. Visualization and annotation of the structural models, including color-coding of individual proteins and highlighting of the PhoU Ala147 residue, were performed using PyMOL (Schrödinger, LLC).

### Membrane vesicle preparation

Cells were grown in N-minimal medium containing 10 mM phosphate (high Pi) until cells reached an OD_600_ of 0.5. Cells were normalized based on OD_600_ measurements. Crude extracts were prepared in TBS buffer by sonication, and the supernatants were centrifuged for 1 h at 32,000 × g (LaboGene, 1730R). The resulting pellets were resuspended in 50 μl of TBS buffer. The protein concentration in the prepared membrane fractions was determined using a NanoDrop spectrophotometer (Thermo Fisher).

### Measuring autophosphorylation of PhoR histidine kinase

The autophosphorylation of PhoR histidine kinase was measured as previously described (Choi et al., 2019). Membrane vesicles (50 µg) prepared from strains expressing either wild-type PhoU or the PhoU A147E variant were incubated in 100 µl of TBS buffer containing 1 mM MgCl_2_ at room temperature. The reaction was initiated by adding [γ-^32^P] ATP (10 µCi; PerkinElmer), and 10 µl aliquots were collected at the indicated time points. Each aliquot was immediately mixed with 10 µl of 5×SDS loading buffer (Biosesang) to stop the reaction. Samples were incubated at 70°C for 5 min and electrophoresed on a 12% SDS-polyacrylamide gel. Following electrophoresis, gels were dried on Whatman filter paper using a Model 583 gel dryer (Bio-Rad) and analyzed for phosphorylated PhoR using a Typhoon phosphorimager (GE Healthcare). Phosphorylated PhoR bands were identified using samples prepared from wild-type and *phoR* mutant *Salmonella* grown for 5 h in N-minimal medium containing 0.01 mM Pi, a condition known to induce the PhoB/PhoR two-component system.

### Confocal laser scanning microscopy

Cells were grown overnight in N-minimal media containing 10 mM Mg^2+^. A 1:100 dilution of the overnight culture was inoculated into 15 ml of fresh N-minimal medium containing 0.01 mM Mg^2+^ and incubated for 5 h. Bacterial cells were harvested by centrifugation and washed three times with McIlvaine buffer (0.2 M Na_2_HPO_4_ [16.47 ml], 0.1 M citric acid [3.53 ml], pH 7.0). The cells were stained with 50 μg/ml DAPI solution (Sigma-Aldrich, F6057) for 30 min in the dark, followed by a brief wash with UltraPure water. For intracellular polyphosphate quantification, the stained bacteria were transferred to a 96-well plate, and fluorescence was measured using a Synergy microplate reader with excitation at 415 nm and emission at 560 nm. For visualization of polyphosphate granules, DAPI-stained cells were additionally labeled with FM4-64 (Invitrogen, T3166) at a final concentration of 20 μg/ml for 10 min at room temperature. After a brief wash, the stained bacteria were placed onto poly-L-lysine-coated glass slides, fixed, and mounted with Anti-Fade Fluorescence Mounting Medium (Abcam, ab104135). A coverslip (Marienfeld) was placed on top, and the sealed with nail polish. Confocal images were acquired using a Leica SP8X confocal laser scanning microscope (CLSM) with a 100× oil immersion objective. The excitation/emission detection settings were as follows: nucleus (405 nm/420 nm), FM4-64 (515 nm/640 nm), and polyphosphate (405 nm/570 nm).

### Macrophage survival assay

Intracellular survival assays were performed using the J774A.1 murine macrophage-like cell line. A total of 5 × 10^5^ macrophages were seeded in 24-well plates containing Dulbecco’s Modified Eagle’s Medium (DMEM) supplemented with 10% fetal bovine serum (FBS) and incubated at 37°C with 5% CO_2_. Overnight-grown bacteria were added to the macrophages at a multiplicity of infection (MOI) of 10. Plates were centrifuged at 1,000 rpm for 5 min at room temperature and incubated for an additional 30 min to facilitate bacterial uptake. Extracellular bacteria were removed by washing the wells three times with phosphate-buffered saline (PBS), followed by incubation in DMEM supplemented with 10% FBS and 120 μg/ml gentamicin for 1 h to kill remaining extracellular bacteria. To determine intracellular bacterial counts at 1 h post-infection, macrophages were lysed with PBS containing 0.1% Triton X-100, and serial dilutions of the lysates were plated on Luria-Bertani (LB) agar. For 21 h time points, the medium was replaced after the initial 1 h with fresh DMEM containing 12 μg/ml gentamicin, and incubation continued at 37°C. After 21 h, cells were lysed as above, and viable intracellular bacteria were quantified by plating. Percentage survival was calculated by dividing colony-forming units (CFUs) recovered at 21 h by those recovered at 1 h. All experiments were performed in duplicate and repeated independently at least three times.

### Mouse virulence assay

Six- to eight-week-old female C3H/HeN mice were inoculated intraperitoneally with approximately 10^3^ colony-forming units (CFU) of *Salmonella* strains. Mouse survival was monitored for 21 days. Virulence assays were repeated three times with consistent outcomes, and the presented data represent groups of five mice per strain. All animals were housed in a temperature- and humidity-controlled facility with a 12 h light/12 h dark cycle. All experimental procedures were conducted in accordance with protocols approved by the Institutional Animal Care and Use Committee (IACUC) of Kangwon National University (approval no. KW-210728-1).

## Results

### The Ala147 residue of *Salmonella* PhoU is required for interaction with PhoR histidine kinase

**Fig. 2.**
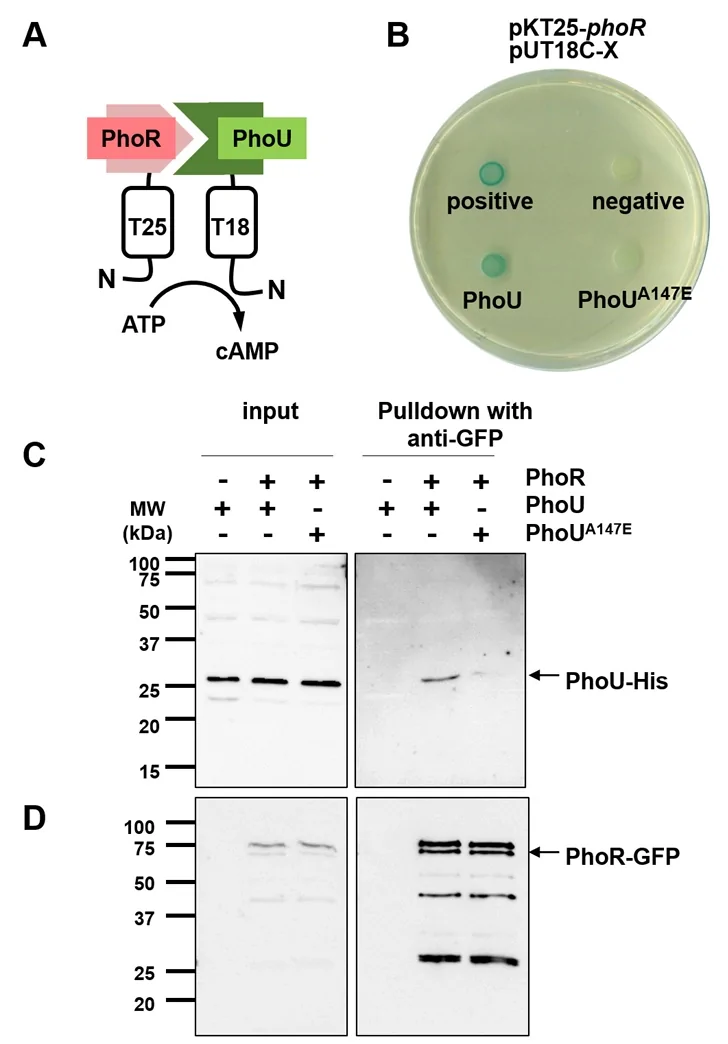
PhoU Ala147 is required for interaction with PhoR. (A) Schematic representation of the bacterial two-hybrid assay used to assess the interaction between PhoR and either wild-type PhoU or the PhoU^A147E^ mutant. (B) *Escherichia coli* BTH101 cells were co-transformed with plasmid combinations expressing N-terminal fusions of the *cyaA* T18 fragment to wild-type *phoU* (pUT18C-*phoU*) or the A147E variant (pUT18C-*phoU*^A147E^), and C-terminal fusions of the *cyaA* T25 fragment to *phoR* (pKT25-*phoR*), or with the empty vector (pUT18C) as a negative control. Cells co-expressing pUT18-*mgtC* and pKT25-*mgtR* served as a positive control. Spotted cultures were incubated on LB agar plates supplemented with 0.5 mM IPTG and 80 μM X-Gal at 30°C for 48 h. Blue colonies indicate a positive protein–protein interaction. (C–D) The C-terminally GFP-tagged PhoR protein immunoprecipitates PhoU. (Left) Crude lysates prepared from wild-type *Salmonella* strains coexpressing PhoR-GFP and either PhoU-His or PhoU^A147E^-His were analyzed by immunoblotting with anti-His (C) and anti-GFP (D) antibodies. (Right) Eluted fractions from the same strains were subjected to immunoprecipitation using anti-GFP antibody-coated beads and analyzed by immunoblotting with anti-His (C) and anti-GFP (D) antibodies.

In *E. coli*, PhoU binds to the PAS domain of PhoR, leading to the suppression of *pho* regulon gene expression under high phosphate conditions (Gardner et al., 2014; Muda et al., 1992). The Ala147 and Arg 148 residues in PhoU were proposed to be critical for this interaction in the previous genetic analysis and protein modeling in *E. coli* (Gardner et al., 2015; Muda et al., 1992; Torriani and Rothman, 1961). In addition, amino acid sequence alignment showed that these residues are conserved in PhoU from *E. coli* and *Salmonella enterica* (Choi et al., 2022). To determine whether these residues in *Salmonella* PhoU are similarly involved in binding PhoR histidine kinase, we performed a bacterial two-hybrid assay using a strain coexpressing T18C-fused wild-type PhoU or the PhoU A147E variant and PhoR-T25 (Fig. 2A). Coexpression of T18C-fused wild-type PhoU and PhoR-T25 exhibited a blue color on LB medium containing X-gal, indicating a physical interaction between PhoU and PhoR (Fig. 2B). In contrast, the PhoU A147E variant did not exhibit a color (Fig. 2B), demonstrating that the Ala147 residue in *Salmonella* PhoU is essential for PhoR interaction.

Protein modeling suggests that PhoU interacts with the PAS domain of PhoR and is in close proximity to the base of PstB in the PstSCAB_2_ phosphate transporter (Fig. S1). In this model, the PhoU Ala147 residue is oriented toward the PAS domain of PhoR but does not appear to contact PstB. As predicted, the PhoU A147E substitution weakened the interaction with PhoR (Fig. S1C and S1E) but did not affect the interaction with PstB (Fig. S1D and S1F). As a control, we chose the PhoU A147K substitution, which was previously reported to have no effect on the interaction (Choi et al., 2022). Since the *phoU* R148A substitution also maintained the interactions with PhoR and PstB, the R148 residue appears to be less important for the PhoU-PhoR PAS domain interaction, unlike in *E. coli* (Fig. S1C and S1D) (Gardner et al., 2015).

The PhoU-PhoR interaction was further confirmed by immunoprecipitation. A C-terminally GFP-tagged PhoR protein successfully immunoprecipitated C-terminally His-tagged wild-type PhoU (Fig. 2C and 2D). Similarly to the two-hybrid assay, the same PhoR protein failed to immunoprecipitate the PhoU A147E variant (Fig. 2C and 2D), indicating that PhoU Ala147 residue is necessary for PhoR interaction.

### The *phoU*^A147E^ substitution derepresses mRNA and protein levels of *pho* genes in high phosphate

**Fig. 3.**
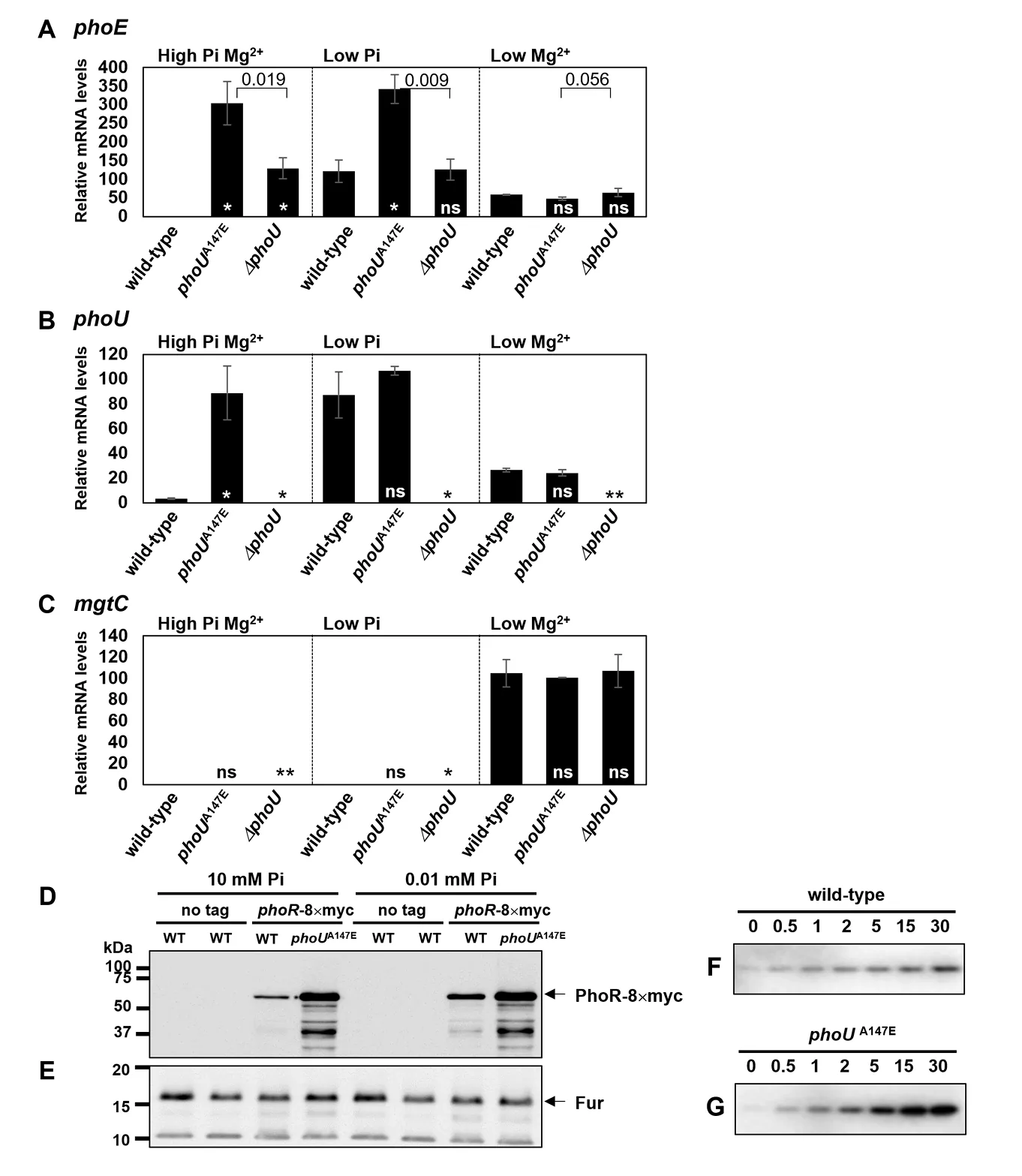
The *phoU*^A147E^ substitution derepresses *pho* regulon gene expression under high-phosphate conditions. (A–C) Relative mRNA levels of *phoE* (A), *phoU* (B), and *mgtC* (C) were measured in *Salmonella* strains carrying wild-type *phoU* (14028s), the A147E mutant allele (SM323), or a ∆*phoU* deletion (SM101). Bacteria were grown for 5 h in N-minimal medium supplemented with 10 mM Pi and 10 mM Mg^2+^ (High Pi Mg^2+^), 10 mM Mg^2+^ and 0.01 mM Pi (Low Pi), or 10 mM Pi and 0.01 mM Mg^2+^ (Low Mg^2+^). Data represent means ± SD from three independent experiments (n = 3). Relative mRNA levels were calculated as (target RNA / *rrsH* RNA) × 10^4^. Statistical significance between wild-type and corresponding mutant strains was assessed using a paired two-tailed *t*-test; ns = not significant, p^*^ < 0.05, p^**^ < 0.01. (D–E) The *phoU*^A147E^ substitution derepresses PhoR protein levels under high-phosphate conditions. Western blot analysis of crude extracts from strains carrying wild-type *phoU* (SM454), the *phoU*^A147E^ mutation (SM457), or an untagged control strain (14028s), all in a background with C-terminally 8×Myc-tagged *phoR*, except for the no-tag control. Blots were probed with anti-Myc (D) to detect PhoR-8×Myc and with anti-Fur (E) as a loading control. Bacteria were grown for 5 h in N-minimal medium supplemented with either 10 mM or 0.01 mM inorganic phosphate (Pi), as described in 'Materials and Methods'. (F–G) Autophosphorylation assay of *Salmonella* strains carrying either wild-type *phoU* (F) or the *phoU* A147E substitution (G), performed with [γ-^32^P] ATP at the indicated times (min). Bacteria were grown for 5 h in N-minimal medium supplemented with 10 mM Pi, and the membrane vesicles were prepared as described in 'Materials and Methods'.

We then investigated the consequence of the PhoU A147E substitution, which disrupts PhoR interaction. Because PhoU primarily functions as a negative regulator of PhoR kinase activity in high phosphate, we measured the mRNA levels of *phoE*, a PhoB-dependent gene encoding the PhoE phosphoporin, in both high- and low-phosphate media. In wild-type, *phoE* mRNA was undetectable in high phosphate but was strongly induced in low phosphate (Fig. 3A). In contrast, the *phoU* A147E substitution strongly elevated *phoE* mRNA levels in both high and low phosphate media (Fig. 3A). A similar expression pattern was detected in the *phoU* deletion mutant, indicating that the disruption of the PhoU-PhoR interaction via the A147E substitution results in constitutive *phoE* expression in high phosphate media. The *phoU* gene itself is also controlled by the PhoB/PhoR two-component system. Thus, *phoU* mRNA levels in the wild-type and the *phoU* A147E mutant were comparable to those of *phoE*, whereas no *phoU* transcript was detected in the *phoU* deletion mutant (Fig. 3B).

In contrast, the *phoU* A147E substitution did not affect expression patterns of the *phoE* and *phoU* genes in low Mg^2+^ (Fig. 3A–3C), suggesting the *phoU* A147E substitution affects phosphate signaling but not low Mg^2+^-mediated signaling. As a control, mRNA levels of *mgtC* encoding a virulence protein were highly induced in all above strains in low Mg^2+^ (Fig. 3C) (Alix and Blanc-Potard, 2007; Lee and Lee, 2015).

Additionally, when we measured PhoR protein levels using a strain with a C-terminally myc-tagged *phoR* gene, the *phoU* A147E substitution elevated PhoR protein levels in both high and low phosphate media (Fig. 3D and 3E). The elevated expression of *phoE* and *phoU* mRNA or PhoR protein levels of the *phoU* A147E mutant in high phosphate may result from the fact that the A147E substitution disrupts both the PhoU-PhoR PAS domain interaction and the associated suppression of PhoR autophosphorylation activity. Indeed, when we measured the rate of PhoR autophosphorylation in membrane vesicles prepared from the wild-type and *phoU* A147E mutant, the A147E substitution increased the rate of PhoR autophosphorylation compared to the wild-type in high phosphate (Fig. 3F and 3G). Collectively, these data demonstrate that the PhoU Ala147 residue is required for its interaction with the PhoR histidine kinase and for the repression of PhoR autophosphorylation and PhoB-dependent gene expression in high phosphate.

### PhoU Ala147-mediated interaction with PhoR is required for *Salmonella* virulence

Maintaining phosphate (Pi) homeostasis is essential for *Salmonella* virulence, as Pi concentrations are proposed to be low within the *Salmonella*-containing vacuole during infection (García-del Portillo et al., 2008; Röder et al., 2021). In agreement with this notion, the *phoU* deletion mutant exhibited attenuated virulence in *Salmonella* (Choi et al., 2022).

**Fig. 4.**
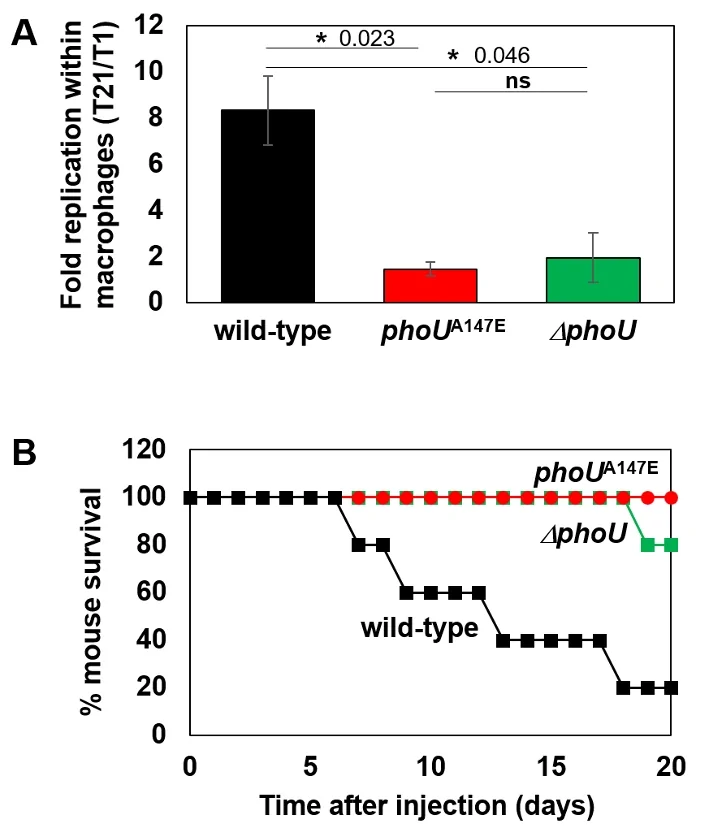
PhoU Ala147-mediated interaction with PhoR is required for *Salmonella* virulence. (A) Substitution of Ala147 with Glu in PhoU reduces intracellular survival in J774A.1 macrophages. Wild-type *Salmonella* (14028s), a chromosomal *phoU*^A147E^ mutant (SM323), and a *phoU* deletion mutant (SM101) were used to infect macrophages, and bacterial counts were determined at 1 h (T1) and 21 h (T21) post-infection. Fold replication was calculated as [CFU at T21 / CFU at T1]. Data represent means ± SD from three independent infections. Statistical significance between strains was assessed using a paired two-tailed *t*-test; ns = not significant, p^*^ < 0.05. (B) The *phoU*^A147E^ mutation also decreases virulence in mice. Survival of C3H/HeN mice (n = 5 per group) inoculated intraperitoneally with ~10^3^ CFU of the strains listed above was monitored over 21 days. According to the log-rank test, there were significant differences among three groups (*P* < 0.0001).

Given that the *phoU* A147E mutant shows similar derepression of *pho* regulon genes as the *phoU* deletion mutant, we wondered whether the *phoU* A147E substitution also affects *Salmonella* pathogenesis. When we infected wild-type *Salmonella* strain into J774A.1 macrophage-like cells, intracellular replication of wild-type *Salmonella* increased approximately 8-fold by 21 h post-infection (Fig. 4A). In contrast, the *phoU* A147E substitution mutant showed decreased replication, reaching only ~20% of wild-type levels (Fig. 4A). This decrease in macrophage replication was similar to that of the *phoU* deletion mutant, indicating that the macrophage survival phenotype of the *phoU* A147E mutant resemble that of the *phoU* deletion mutant, consistent with its failure to repress *pho* regulon expression in high phosphate.

Consistent with macrophage survival, when we injected ~3,000 CFU of *Salmonella* strains into C3H/HeN mice, the *phoU* A147E substitution resulted in a complete loss of mouse virulence, with 100% survival of infected mice, compared to only 20% survival in mice infected with wild-type *Salmonella* (Fig. 4B). Similarly, the *phoU* deletion mutant also exhibited a defect in mouse virulence, with 80% of mice surviving 20 days post-infection (Fig. 4B). Together, these results demonstrate that both the *phoU* A147E substitution and *phoU* deletion impair intramacrophage survival and attenuate mouse virulence. In conclusion, these findings suggest that the interaction between PhoU Ala147 and the PAS domain of PhoR is essential for *Salmonella* virulence and for repression of *pho* regulon expression in high phosphate.

### The *phoU* Ala147-to-Glu substitution does not exhibit a growth defect regardless of phosphate concentration

**Fig. 5.**
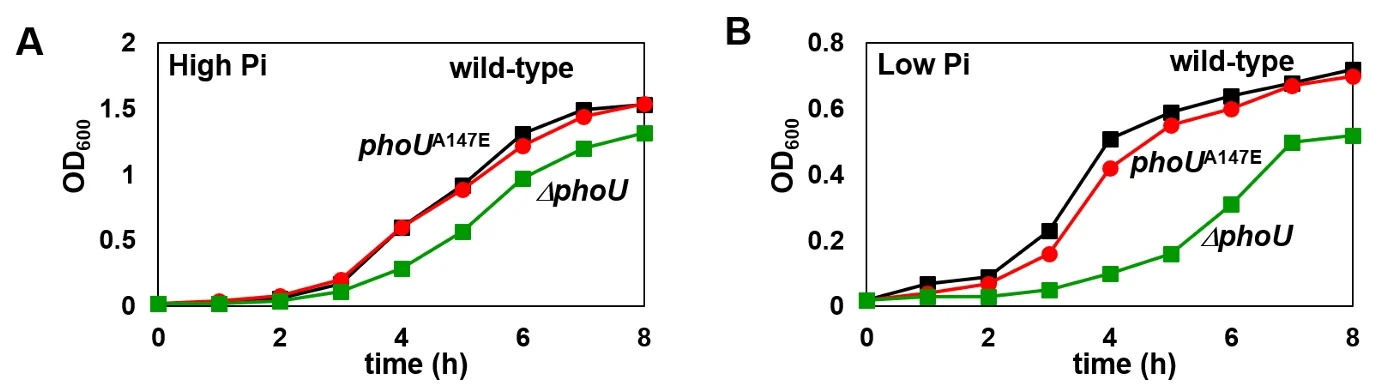
The *phoU*^A147E^ substitution does not impair bacterial growth under either high- or low-phosphate conditions. (A, B) Growth curves of *Salmonella* strains 14028s (wild-type), SM323 (*phoU*^A147E^), and SM101 (Δ*phoU*). Bacteria were cultured in N-minimal medium containing 10 mM Pi (A) or 0.01 mM Pi (B) at 37°C for 8 h. Optical density at 600 nm (OD_600_) was measured hourly.

However, not all phenotypes of the *phoU* deletion mutant were recapitulated by the *phoU* A147E substitution mutant. First, the *phoU* deletion mutant forms small colonies on LB solid agar but the colony size of the *phoU* A147E mutant was similar to that of wild-type. When we measured a growth curve in N-minimal media containing high phosphate, the *phoU* A147E substitution mutant grew similarly to wild-type, whereas the *phoU* deletion mutant exhibited delayed growth in the same media (Fig. 5A). In N-minimal media containing low phosphate, the growth defect of the *phoU* deletion mutant was exaggerated (Fig. 5B). However, even in this condition, the *phoU* A147E substitution mutant grew similarly to wild-type, indicating that disruption of the PhoU-PhoR PAS domain interaction derepresses *pho* regulon gene expression in high phosphate but does not lead to a growth defect (Fig. 5B). This represents the first report of a *phoU* mutation that uncouples the growth defect phenotype from the derepression of *pho* gene expression in high phosphate.

### The *phoU* Ala147-to-Glu substitution does not lead to in vivo polyphosphate accumulation

**Fig. 6.**
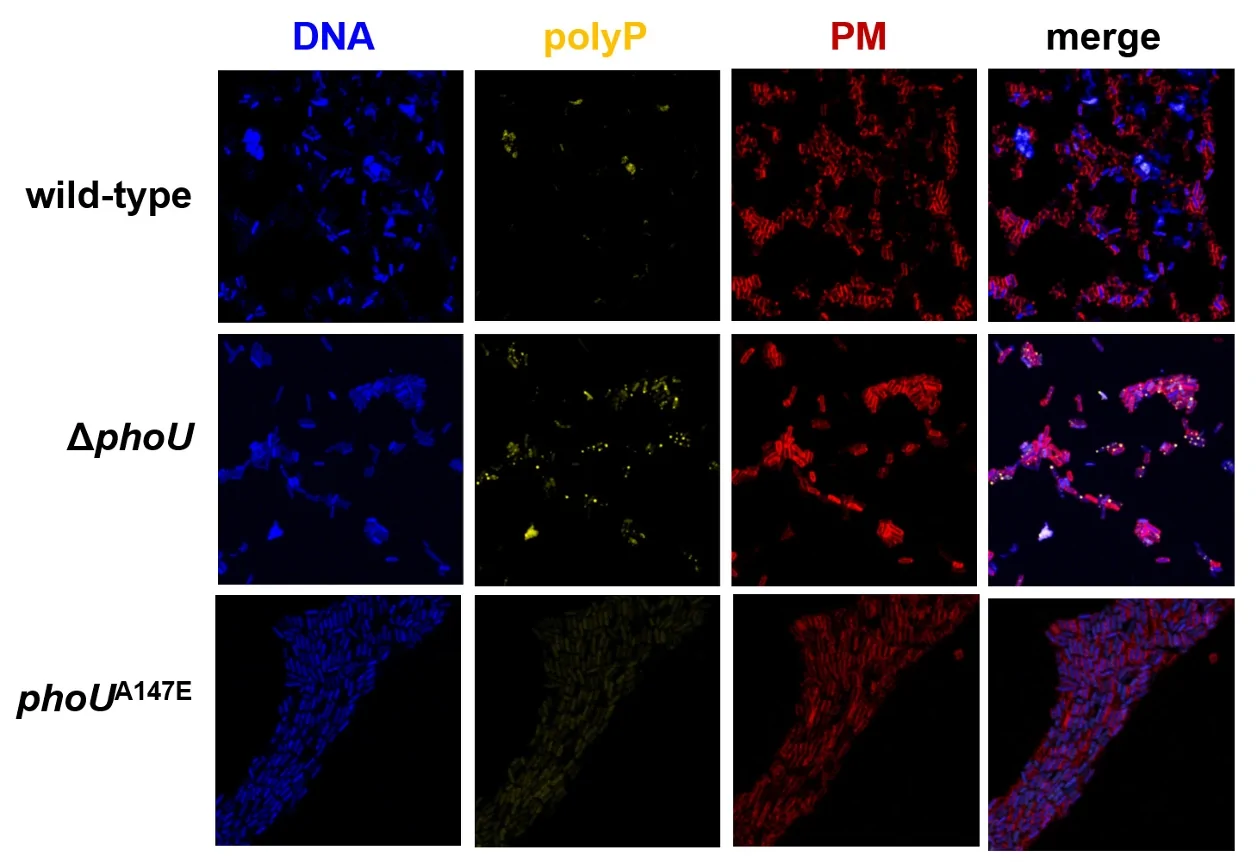
The *phoU*^A147E^ substitution does not affect in vivo polyphosphate accumulation. Confocal laser scanning microscopy (CLSM) images of wild-type *Salmonella* (14028s), the chromosomal *phoU*^A147E^ mutant (SM323), and the *phoU* deletion mutant (SM101). Bacteria were grown for 5 h in N-minimal medium containing 0.01 mM Mg^2+^. Polyphosphate granules and nucleoids were stained with DAPI, and plasma membranes were stained with FM4-64, as described in ‘Materials and Methods.’ Merged fluorescence images are shown to visualize polyphosphate localization. polyP: polyphosphate; PM: plasma membrane.

Polyphosphate (polyP) is a linear polymer of phosphate units linked by high-energy phosphoanhydride bonds. In bacteria, polyP is involved in diverse physiological processes, such as energy storage, stress responses, antibiotic resistance, and virulence (Kornberg et al., 1999; Rao et al., 2009). Previous studies have reported the accumulation of polyphosphate in *phoU* mutants in many bacterial species (de Almeida et al., 2015; Li and Zhang, 2007; Lubin et al., 2016; Morohoshi et al., 2002). To access whether the *phoU* A147E substitution affects polyphosphate accumulation in *Salmonella*, we used confocal laser scanning microscopy (CLSM) to visualize polyP granules. As expected, the *phoU* mutant displayed strong signals from polyP granule staining compared to the wild-type strain (Fig. 6). However, the *phoU* A147E substitution mutant did not exhibit detectable polyphosphate granule signals, even lower than that of the wild-type (Fig. 6). These data indicate that the *phoU* A147E substitution does not lead to polyphosphate accumulation in *Salmonella*. Collectively, disruption of PhoR PAS-PhoU interaction via PhoU A147E substitution results in derepression of *pho* regulon gene expression and attenuation of *Salmonella* virulence, but does not affect phenotypes such as growth defects or polyphosphate accumulation.

## Discussion

Using bacterial two-hybrid assay and immunoprecipitation (Fig. 2), we identified that PhoU interacts with the PAS domain of PhoR via Ala147 in *Salmonella*, consistent with a prior study conducted in *E. coli* (Gardner et al., 2015). The introduction of the *phoU* Ala147-to-Glu substitution in *Salmonella* disrupts this interaction, leading to increased *phoE* mRNA levels even under high phosphate conditions (Fig. 3). This phenotype supports the role of that PhoU as a negative regulator of the *phoU* regulon via binding to the PhoR PAS domain. Additionally, the increased protein level of PhoR in *phoU* Ala147-to-Glu mutant under high phosphate conditions further highlights the importance of Ala147 in repressing *pho* regulon gene expression (Fig. 3). Together, the constitutive expression in *phoE* and elevated PhoR levels due to the Ala147-to-Glu substitution suggest that PhoU binding to the PhoR PAS domain via Ala147 suppresses *pho* regulon gene expression, likely by inhibiting auto-phosphorylation activity of PhoR. In this aspect, the *phoU* A147E substitution phenocopies the *phoU* deletion mutant, indicating that disruption of the PhoU-PhoR PAS domain interaction is sufficient to explain phenotypes of the *phoU* deletion mutant. In agreement with this notion, the *phoU* A147E mutant completely attenuated *Salmonella* virulence in mice, similar to the *phoU* deletion mutant (Fig. 4).

Interestingly, however, not all phenotypes of the *phoU* deletion mutant were recapitulated in the *phoU* A147E substitution mutant. While the *phoU* deletion mutant showed a growth defect and accumulated polyphosphate granules, the A147E substitution did not impair growth or induce polyP accumulation in *Salmonella* (Figs. 5 and 6). This suggests that the growth defect and polyphosphate accumulation phenotypes in the *phoU* mutant are not linked to the PhoU-PhoR PAS interaction, and that the phenotypes associated with *phoU* deletion can be genetically dissected into distinct groups.

Previous studies have shown that PhoU directly interacts with PstB in the PstSCAB_2_ transporter (Gardner et al., 2014), and this interaction may inhibit phosphate uptake (diCenzo et al., 2017). Our structure modeling of the PhoR-PhoU-PstSCAB_2_ complex, along with two-hybrid assays between PhoU and PstB (Fig. S1E), support this interaction. Although disruption of the PhoU-PhoR PAS domain interaction elevates *pho* regulon gene expression and the production of *pho* regulon components such as the PstSCAB_2_ transporter and PhoU, the interaction between PhoU and PstB is still maintained. Thus, this is expected to suppress the activity of the PstSCAB_2_ transporter in both high and low phosphate conditions. In agreement with this idea, it was previously reported that the *E. coli* *phoU35* mutant – which was later identified to carry the same substitution in *phoU* – did not show an altered rate of Pi incorporation compared to the wild-type in phosphate-depleted conditions (Gardner et al., 2015; Muda et al., 1992). Because the *phoU* A147E substitution maintains the PhoU-PstB interaction and does not result in polyphosphate accumulation, this suggests that relieving suppression of phosphate uptake activity might contribute to the polyphosphate accumulation phenotype. Interestingly, the *phoU* A147E mutant exhibited even higher levels of *phoE* mRNA and PhoR protein than the *phoU* deletion mutant (Fig. 3). Since the A147E substitution differs from the *phoU* deletion in that it disrupts the PhoU-PhoR interaction while maintaining the PhoU-PstB interaction within the PhoR-PhoU-PstSCAB_2_ complex, these differences in expression levels appear to result from altered feedback regulation: loss of PhoR interaction prevents suppression of PhoR kinase activity or switching to phosphatase activity, while maintenance of the PstB interaction may limit phosphate uptake in high phosphate conditions. These mechanisms remain to be further elucidated.

Our previous work demonstrated that *Salmonella*’s PhoU can interact with PhoR histidine kinase at two distinct sites: the PAS and CA domains (Choi et al., 2022). The interaction with the PhoR CA domain, mediated by Arg184 residue of PhoU, is required for the activation of PhoR in low Mg^2+^ (Choi et al., 2022), a condition physiologically relevant during host infection. Thus, the PhoU-PhoR CA domain interaction is necessary for *Salmonella* virulence (Choi et al., 2022). Here, we show that the substitution of Ala147 with Glu in PhoU, which disrupts the PhoU-PhoR PAS interaction, also attenuates virulence in mouse, suggesting that this interaction likewise contributes to *Salmonella* pathogenicity (Fig. 4). Notably, the *phoU* A147E substitution does not impair bacterial growth in low Mg^2+^, a condition commonly used to model infection-relevant conditions, providing an example that uncouples growth in low Mg^2+^ from in vivo virulence. Since both PhoU-PhoR interactions-one inhibitory (PAS) and one activating (CA)-are required for full virulence, this highlights the importance of fine-tuning *pho* regulon expression in *Salmonella* pathogenesis.

This study provides offers a new perspective on the physiological roles of PhoU in bacteria. The increase in antibiotic and secondary metabolite production observed in *Streptomyces* (Martin-Martin et al., 2017; Tang et al., 2022) appears to be linked to the PhoB-PstB interaction, as polyphosphate (polyP) accumulation is a key regulatory factor. Similarly, the reduced persister formation and antibiotic resistance in the *phoU* deletion mutant (Wang et al., 2013, 2017), which are associated with intracellular phosphate levels, suggest that these phenotypes are mediated by PhoU’s interaction with the PstSCAB_2_ transporter. Furthermore, in *Caulobacter crescentus*, polyphosphate accumulation occurs independently of the PhoB/PhoR system (Lubin et al., 2016), likely due to mis-regulation of the Pst system. In conclusion, targeted amino acid substitutions, rather than complete deletion of *phoU*, allows dissection of the complex regulatory roles of PhoU in *pho* regulon control and phosphate homeostasis. These findings highlight the complexity of PhoU-mediated regulation and PhoU’s role in coordinating multiple physiological pathways in bacteria.

## Supplementary Information

Table S1. Bacterial strains, plasmids, and oligonucleotides used in this study

Fig. S1. PhoU Ala147 is not required for interaction with PstB. (A–B) Schematic representation of the bacterial two-hybrid assay used to assess interactions between PhoR or PstB and PhoU or its variants. (C–D) *Escherichia coli* BTH101 strains were co-transformed with plasmid pairs: pKT25 derivatives expressing a C-terminal fusion of the *cyaA* T25 fragment to *phoR* (C) or *pstB* (D), and pUT18 or pUT18C derivatives expressing N-terminal fusions of the *cyaA* T18 fragment to wild-type *phoU* or C-terminal fusions to *phoU*^A147E^, *phoU*^A147K^, or *phoU*^R148A^. Cells containing pUT18C served as a negative control. Cells co-expressing pUT18-*mgtC* and pKT25-*mgtR* served as a positive control. Cultures were spotted onto LB agar plates containing 80 μM X-Gal and 0.5 mM IPTG and incubated at 30°C for 48 h. Blue colonies indicate positive interactions. (E–F) Alphafold-assisted modeling of PhoR-PhoU-Pst complex. PhoU (UniProt: A0A0F6B922) and its interacting partners PhoR (A0A0F6AXL9) and PstB (A0A0F6B923) in *Salmonella* Typhimurium 14028s, were used to generate complex prediction. PhoU is shown in green, with Ala147 highlighted in red. PhoR and PstB are shown in pink and sky blue, respectively.
