# Supplementary material for: PhoU interaction with the PhoR PAS domain is required for repression of the pho regulon and Salmonella virulence, but not for polyphosphate accumulation
Source: J Microbiol. 2025 Sep 30;63(9):e2505013. doi: 10.71150/jm.2505013 (PMC13577022; doi:10.71150/jm.2505013)
Supplement: Supplementary file 1 [file jm-2505013-Supplementary-Fig-S1.pdf]

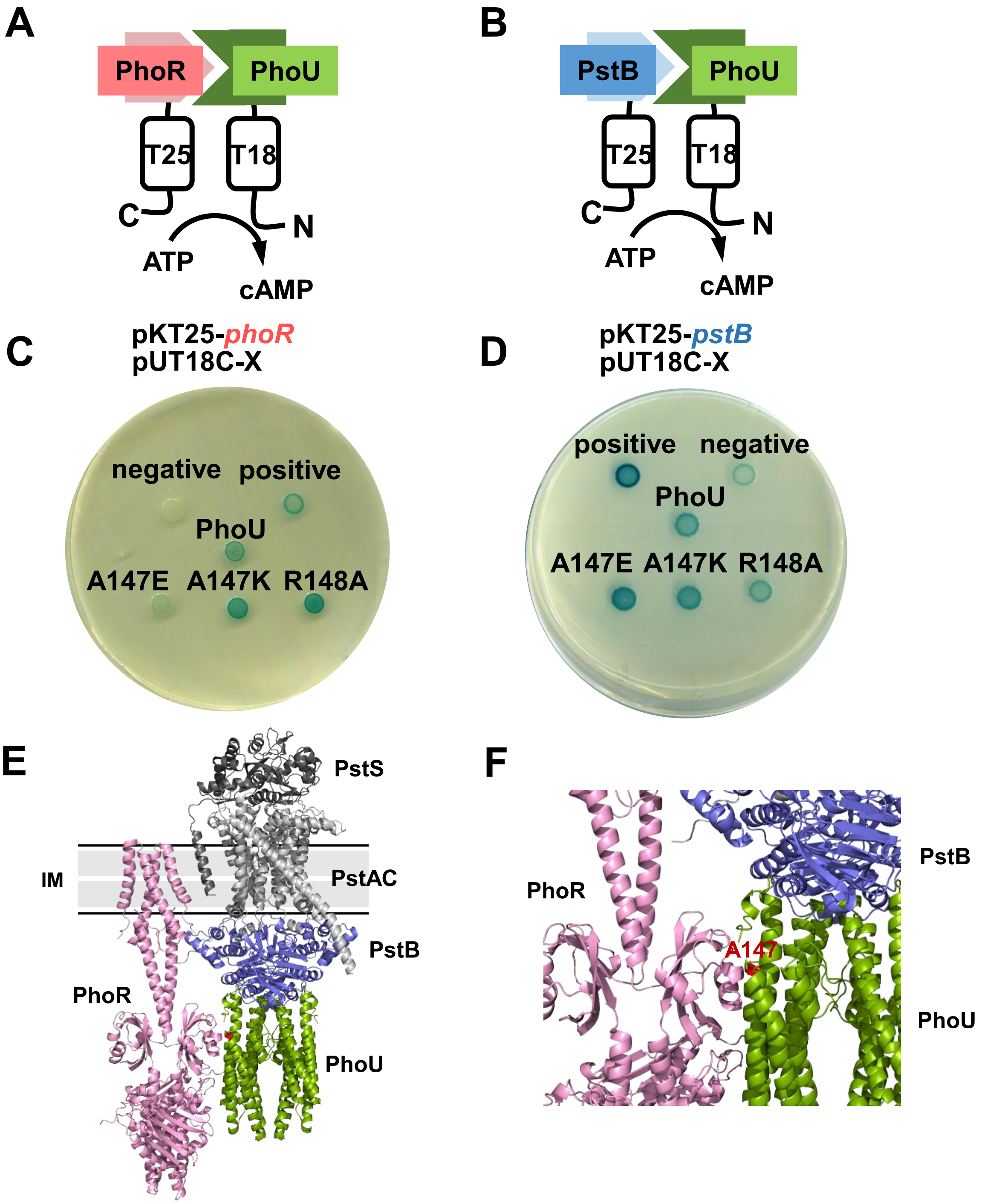

Fig. S1. PhoU Ala147 is not required for interaction with PstB. (A–B) Schematic representation of the bacterial two-hybrid assay used to assess interactions between PhoR or PstB and PhoU or its variants. (C–D) *Escherichia coli* BTH101 strains were co-transformed with plasmid pairs: pKT25 derivatives expressing a C-terminal fusion of the *cyaA* T25 fragment to *phoR* (C) or *pstB* (D), and pUT18 or pUT18C derivatives expressing N-terminal fusions of the *cyaA* T18 fragment to wild-type *phoU* or C-terminal fusions to *phoU*^{A147E}, *phoU*^{A147K}, or *phoU*^{R148A}. Cells containing pUT18C served as a negative control. Cells co-expressing pUT18-*mgtC* and pKT25-*mgtR* served as a positive control. Cultures were spotted onto LB agar plates containing 80 μM X-Gal and 0.5 mM IPTG and incubated at 30°C for 48 h. Blue colonies indicate positive interactions. (E–F) Alphafold-assisted modeling of PhoR-PhoU-Pst complex. PhoU (UniProt: A0A0F6B922) and its interacting partners PhoR (A0A0F6AXL9) and PstB (A0A0F6B923) in *Salmonella* Typhimurium 14028s, were used to generate complex prediction. PhoU is shown in green, with Ala147 highlighted in red. PhoR and PstB are shown in pink and sky blue, respectively.
