# Supplementary material for: PhoU interaction with the PhoR PAS domain is required for repression of the pho regulon and Salmonella virulence, but not for polyphosphate accumulation
Source: J Microbiol. 2025 Sep 30;63(9):e2505013. doi: 10.71150/jm.2505013 (PMC13577022; doi:10.71150/jm.2505013)
Supplement: Supplementary file 2 [file jm-2505013-Supplementary-Table-S1.pdf]

Table S1. Bacterial strains, plasmids, and oligonucleotides used in this study.

(A) Bacterial strains and plasmids

**Name**	**Description**	**Reference**
***S. enteric* serovar Typhimurium**		
14028s	wild-type	Fields et al. (1986)
SM101	*ΔphoU*	Choi et al. (2022)
SM323	*phoU* $^{Ala\ 147\ Glu}$	Choi et al. (2022)
SM108	pBAD33-*phoU*-His, pTGFP	This study
SM109	pBAD33-*phoU*-His, pTGFP-*phoR*	This study
SM343	pBAD33-*phoU* $^{Ala\ 147\ Glu}$-His, pTGFP-*phoR*	This study
SM454	*phoR*-8×Myc	Choi et al. (2022)
SM443	*phoU* $^{Ala\ 147\ Glu}$, *phoR*-8×myc::Km^R	This study
SM457	*phoU* $^{Ala\ 147\ Glu}$, *phoR*-8×myc	This study
Escherichia coli		
DH5α	*fhuA2 lac(del)U169 phoA glnV44 Φ80' lacZ(del)M15 gyrA96 recA1 relA1 endA1 thi-1 hsdR17*.	Taylor, Walker et al. (1993)
SM329	DH5α/pBAD33- *phoU* $^{Ala\ 147\ Glu}$-His	This study
BTH101	*F$^-$, cya-854, recA1, endA1, gyrA96 (Nal^r), thi1, hsdR17, spoT1, rfbD1, glnV44(AS)*	Karimova, et al. (1998)
LJ18	BTH101/pUT18-*mgtC*, pKT25-*mgtR*	Choi et al. (2019)
LJ27	BTH101/pUT18-*mgtC*, pKT25	Choi et al. (2019)
SM089	BTH101/pUT18-*phoU*, pKT25-*phoR*	Choi et al. (2022)
SM314	BTH101/pUT18c-*phoU* $^{Ala\ 147\ Glu}$, pKT25-*phoR*	Choi et al. (2022)
SM315	BTH101/pUT18c-*phoU* $^{Ala\ 147\ Lys}$, pKT25-*phoR*	Choi et al. (2022)
SM311	BTH101/pUT18c-*phoU* $^{Arg\ 148\ Ala}$, pKT25-*phoR*	Choi et al. (2022)
SM210	BTH101/pUT18-*phoU*, pKT25-*pstB*	This study
SM326	BTH101/pUT18c-*phoU* $^{Ala\ 147\ Glu}$, pKT25-*pstB*	This study
SM327	BTH101/pUT18c-*phoU* $^{Ala\ 147\ Lys}$, pKT25-*pstB*	This study
SM328	BTH101/pUT18c-*phoU* $^{Arg\ 148\ Ala}$, pKT25-*pstB*	This study
**Plasmids**		
pBAD33	pACYC184 *ori* Cm^R	Guzman et al. (1995)
pBOP508	repR$_{R6K}$ Ap^R 8×myc FRT Km^R FRT	Cho et al. (2006)
pKD46	rep$_{pSC101}^{ts}$ Ap^R P$_{araBAD}$ γ β exo	Datsenko and Wanner (2000)
pCP20	rep$_{pSC101}^{ts}$ Ap^R Cm^R *cI857* λP$_R$*flp*	Datsenko and Wanner (2000)
pUT18	p$_{lac}$ ColEI *ori* Ap^R	Karimova, et al. (2001)
pUT18c	p$_{lac}$ ColEI *ori* Ap^R	Karimova, et al. (2001)

pKT25	p_{lac} p15A*ori* Km^R	Karimova, et al. (2001)
pTGFP	ColE1 *ori* Ap^R 'gfp	Lee and Groisman (2012)

(B) Primers used in this study.

**Name**	**Sequence (from 5' to 3')***	
**Knockout, deletion, tagging**		
KU452	TGA GCT GGA TAA GCT GCT GGC GGG GAA AGA TCC GAA AGA GAT CGG ATC CAG AAT TCG TGA T	*phoR*-8×Myc Km^R insertion
KU453	GGC CCG GTA AGC GCA GCG CCA CCG GGC AAA AGA ATG AGA TGA GCT CGA TCC GTC GAC C	*phoR*-8×Myc Km^R insertion
**Cloning**		
KHU767	CCC AAG CTT TTA GTG GTG ATG GTG ATG ATG CTC TTT CGG ATC TTT CCC CG	*phoU*-His-HindIII-R
KHU768	GCT CTA GAT AAT TCA GGA GTG CGT AAT GGA CAG TCT GAA CCT TAA	*phoU*-XbaI-F
**For qRT-PCR**		
KHQ015	CGCTGGGAAGCTGAGTTTG	*phoE* qPCR-F
KHQ016	CCAGGCCTCAACATCGTACA	*phoE* qPCR-R
KHQ097	CATATTTCCGGCCAGTTCAAC	*phoU* qPCR-F
KHQ098	CGGTGATAGCGTCAGAAAG	*phoU* qPCR-R
6970	CCAGCAGCCGCGGTAAT	*rrsH* qPCR -F
6971	TTTACGCCCAGTAATTCCGATT	*rrsH* qPCR -R

References

Cho BK, Knight EM, Palsson BO. 2006. PCR-based tandem epitope tagging system for *Escherichia coli* genome engineering. *Biotechniques.* 40: 67–72.

Choi S, Choi E, Cho YJ, Nam D, Lee J, et al. 2019. The *Salmonella* virulence protein MgtC promotes phosphate uptake inside macrophages. *Nat Commun.* 10: 3326.

Choi S, Jeong G, Choi E, Lee EJ. 2022. A dual regulatory role of the PhoU protein in *Salmonella Typhimurium*. *mBio.* 13: e0081122.

Datsenko KA, Wanner BL. 2000. One-step inactivation of chromosomal genes in *Escherichia coli* K-12 using PCR products. *Proc Natl Acad Sci USA.* 97: 6640–6645.

Fields PI, Swanson RV, Haidaris CG, Heffron F. 1986. Mutants of *Salmonella Typhimurium* that cannot survive within the macrophage are avirulent. *Proc Natl Acad Sci USA.* 83: 5189–5193.

Guzman LM, Belin D, Carson MJ, Beckwith J. 1995. Tight regulation, modulation, and high-level expression by vectors containing the arabinose PBAD promoter. *J Bacteriol.* 177: 4121–4130.

Karimova G, Pidoux J, Ullmann A, Ladant D. 1998. A bacterial two-hybrid system based on a reconstituted signal transduction pathway. *Proc Natl Acad Sci USA.* 95:5752–5756.

Karimova G, Ullmann A, Ladant D. 2001. Protein-protein interaction between *Bacillus stearothermophilus* tyrosyl-tRNA synthetase subdomains revealed by a bacterial two-hybrid system. *J Mol Microbiol Biotechnol.* 3:73–82.

Lee EJ, Groisman EA. 2012. Tandem attenuators control expression of the *Salmonella mgtCBR v*irulence operon. *Mol Microbiol.* 86:212–224.

Taylor RG, Walker DC, McInnes RR. 1993. *E. coli* host strains significantly affect the quality of small scale plasmid DNA preparations used for sequencing. *Nucleic Acids Res.* 21:1677–1678.
